# Power to identify exposure‐response relationships in phase IIa pulmonary tuberculosis trials with multi‐dimensional bacterial load modeling

**DOI:** 10.1002/psp4.13089

**Published:** 2023-12-15

**Authors:** Simon E. Koele, Thomas P. C. Dorlo, Caryn M. Upton, Rob E. Aarnoutse, Elin M. Svensson

**Affiliations:** ^1^ Department of Pharmacy, Radboudumc Research Institute for Medical Innovation (RIMI) Radboud University Medical Center Nijmegen The Netherlands; ^2^ Department of Pharmacy Uppsala University Uppsala Sweden; ^3^ TASK Applied Science Cape Town South Africa

## Abstract

Adequate power to identify an exposure‐response relationship in a phase IIa clinical trial for pulmonary tuberculosis (TB) is important for dose selection and design of follow‐up studies. Currently, it is not known what response marker provides the pharmacokinetic‐pharmacodynamic (PK‐PD) model more power to identify an exposure‐response relationship. We simulated colony‐forming units (CFU) and time‐to‐positivity (TTP) measurements for four hypothetical drugs with different activity profiles for 14 days. The power to identify exposure‐response relationships when analyzing CFU, TTP, or combined CFU + TTP data was determined at 60 total participants, or with 25 out of 60 participants in the lowest and highest dosing groups (unbalanced design). For drugs with moderate bactericidal activity, power was low (<59%), irrespective of the data analyzed. Power was 1.9% to 29.4% higher when analyzing TTP data compared to CFU data. Combined analysis of CFU and TTP further improved the power, on average by 4.2%. For a drug with a medium‐high activity, the total sample size needed to achieve 80% power was 136 for CFU, 72 for TTP, and 68 for combined CFU + TTP data. The unbalanced design improved the power by 16% over the balanced design. In conclusion, the power to identify an exposure‐response relationship is low for TB drugs with moderate bactericidal activity or with a slow onset of activity. TTP provides the PK‐PD model with more power to identify exposure‐response relationships compared to CFU, and combined analysis or an unbalanced dosing group study design offers modest further improvement.

**Study Highlights**

**WHAT IS THE CURRENT KNOWLEDGE ON THE TOPIC?**

Identifying an exposure‐response relationship in a phase IIa clinical trial for pulmonary tuberculosis (TB) is important for dose selection and design of follow‐up studies. It is not known what response marker provides the pharmacokinetic‐pharmacodynamic (PK‐PD) model more power to identify an exposure‐response relationship.

**WHAT QUESTION DID THIS STUDY ADDRESS?**

This study investigates the power to identify exposure‐response relationships when analyzing colony‐forming units (CFUs), time‐to‐positivity (TTP), or combined CFU + TTP data, using an in silico PK‐PD approach.

**WHAT DOES THIS STUDY ADD TO OUR KNOWLEDGE?**

The power to identify an exposure‐response relationship is low for TB drugs with moderate bactericidal activity or with a slow onset of activity. TTP is more powerful compared to CFU, and combined analysis or an unbalanced study design offers modest further improvement in power.

**HOW MIGHT THIS CHANGE DRUG DISCOVERY, DEVELOPMENT, AND/OR THERAPEUTICS?**

Our study may improve clinical trial design for phase IIa clinical trials for TB, and may ultimately contribute to more efficient anti‐TB drug development.

## INTRODUCTION

Tuberculosis (TB) is one of the leading causes of the global infectious disease burden. Currently, treatment success rates are ~86% for drug‐susceptible TB and 59% for drug‐resistant TB.^1^ Treatment of TB is lengthy, ranging between 4 and 20 months using a combination of drugs. The development of novel efficacious, safe, and treatment‐shortening drugs is imperative considering the high incidence of drug resistance.^2^ An essential part of anti‐TB drug development is demonstrating bactericidal effect in a phase IIa clinical trial. Although not the primary study objective, additional early knowledge can be gained by powering these studies to evaluate the pharmacokinetic‐pharmacodynamic (PK‐PD) relationship which may better inform later phase trials focused on dose‐finding, and accelerate the drug development timeline.

In these trials, the investigated drug is administered to multiple dosing groups for up to 14 days,^3^, ^4^, ^5^, ^6^ together with a reference standard‐of‐care or monotherapy arm. Usually, 10–15 participants with pulmonary TB are included in each dosing group. The decrease in bacterial load over time, measured by quantitative culture of sputum samples, represents the early bactericidal activity (EBA) of a drug. Currently, the two gold‐standard culture‐based quantification methods of the bacterial load are the enumeration of TB bacilli colony‐forming units (CFUs) on solid media, and the time to positivity (TTP) in liquid media.^7^, ^8^ A better understanding of the dose‐ or exposure‐response relationship in EBA studies may guide dose selection for follow‐up phase IIb or phase III trials, or to deselect a compound with poor EBA. Additionally, exposure‐response data can also aid in identifying patient populations that may benefit most from a particular drug, avoiding ineffective treatments in nonresponsive populations. Therefore, adequate power to identify the dose‐ or exposure‐response relationship is important. However, the quantification of the bacterial load is associated with high inter‐ and intrasubject variability, complicating the determination of a relationship.^9^ The exposure‐response relationship is generally preferred over the dose–response, as the exposure‐response relationship accounts for variability in response due to interindividual differences in PKs.

Model‐based analysis of phase IIa clinical trial data has shown a significant increase in power to identify the activity of a drug compared to traditional statistical analyses.^10^ A study on the combined analysis of CFU and TTP, using a semimechanistic pharmacometric model, showed that both metrics can be combined to evaluate the exposure‐response relationship in a phase IIa trial.^11^ However, it is not known which culture method, or a combination of both methods, may provide the model with more power to identify an exposure‐response relationship. Furthermore, we hypothesized that participants in extreme dose groups contribute more information on the exposure‐response relationship compared to participants in middle dose groups, hence an uneven distribution of participants might improve the power to identify an exposure‐response relationship over a balanced distribution.

Therefore, we investigated the effect of different culture methods, and unbalanced sample sizes on the power to identify exposure‐response relationships in phase IIa trials for patients with pulmonary TB, using in silico clinical trial simulations.

## METHODS

We performed an in silico study, simulating CFU and TTP measurements as part of a virtual phase IIa clinical trial to determine the power to identify exposure‐response relationships. Four hypothetical drugs with similar, dose‐proportional PKs, but different exposure‐effect relationships were virtually administered for 14 days. Duplicate CFU and TTP measurements were simulated on days 0–5 and days 7, 9, and 14 based on individual exposure metrics. Monte‐Carlo mapped power (MCMP) analysis was performed to assess the power to identify an exposure‐response relationship in each simulated clinical trial.^12^

### Pharmacodynamic model

The assumptions for the PD simulation model were based on HIGHRIF1 clinical trial data. In summary, the PANACEA HIGHRIF1 clinical trial investigated high‐dose rifampicin from 10 to 50 mg/kg daily in separate groups of patients with sputum‐smear‐positive pulmonary TB.^6^, ^13^ Rifampicin monotherapy was administered for 7 days, followed by an additional 7 days in which standard‐dose isoniazid, pyrazinamide, and ethambutol were co‐administered. Sputum samples were taken on days 0, 1, 2, 3, 4, 5, 6, 7, 9, and 14, and cultured in duplicate using CFU and TTP quantification methods.

A linear model, a bilinear model with an estimated node, a smooth bilinear model, and a semimechanistic model coupling the bacterial load to TTP were evaluated to describe the decrease in bacterial load over time.^14^, ^15^ The bilinear model with an estimated node provided the best fit. The developed bilinear model simultaneously analyzed CFU and TTP data and was characterized by a shared node parameter, correlated interindividual variation (IIV) for intercepts and slopes (killing rates), and a correlated residual error model. Individual baseline bacterial load and slope were assumed to be log‐normally distributed. The stochastic model included IIV on the baseline bacterial load and slopes, as well as additive residual variability. The M3 method was used to account for the lower limit of quantification and upper limit of quantification observations for CFU and TTP observations, respectively.^16^ The lower limit employed for CFU was 1 Log10(CFU*mL^−1^) sputum and the upper limit of quantification for TTP was Log10(42 days). A baseline chance of obtaining a negative culture result, reflecting the sensitivity of the culture method, was imputed as the percentage of negative culture results during the first 3 days of treatment. A replication error was estimated for the duplicate CFU and TTP measurements. The model fit was evaluated using the objective function value, goodness of fit plots, and visual predictive checks.

### Pharmacokinetic model

Four hypothetical drugs with similar PKs but different exposure‐effect profiles were administered in a daily dose, or double, triple, or four‐fold the daily dose (1–4× dose) for 14 days. A one‐compartmental PK model was used to generate a range of exposures. Typical virtual patients were assumed to have a volume of distribution of 40 L and a clearance of 10 L/h with an IIV coefficient of variation of 30%. No covariate relationships were included in the model.

### Exposure‐response relationship

The developed bilinear PD model (Equation 1) and PK model were subsequently coupled using a maximum effect (*E*_max_) model to determine the individual killing rates for CFU and TTP. The slope parameters 1 and 2 in the PD model were influenced by exposure following an *E*_max_ model using the individual daily area under the simulated drug concentration‐time curve (AUC_0‐24h_), as this exposure metric is commonly used in exposure‐response determination in phase IIa clinical trials (Equation 2).^17^, ^18^

(1) $$\begin{array}{c} \text{Bacterial load}\;\left(t\right)=\text{Intercept}-\text{Slop}e_{1}*t+\epsilon \;t<\text{node}\\ \text{Bacterial load}\;\left(\text{node}\right)=\text{Intercept}-\text{Slop}e_{1}*\text{node}+\epsilon \;t=\text{node}\\ \text{Bacterial load}\;\left(t\right)=\text{Bacterial load}\;\left(\text{node}\right)-\text{Slop}e_{2}*\left(t-\text{node}\right)+\epsilon \;t>\text{node} \end{array}$$

(2) $${\text{Slope}}_{E\max,i}=\frac{\left(E_{\max,i}*\mathrm{AU}C_{0-24}\right)}{\left({\mathrm{EC}}_{50}+\mathrm{AU}C_{0-24}\right)}*\left(1+\text{IIVslop}e_{i}\right)$$

where Slope_*E*max_ represents slopes following an *E*_max_‐relationship with drug exposure applied in Equation 1, and *i* represents either the first or second slope. AUC_0‐24_ represents the individual daily area under the simulated drug concentration‐time curve, and the EC_50_ the exposure needed to achieve 50% of the *E*_max_ on the slope. IIVslope is the inter‐individual variability on the slope parameter.

Drug A was selected to result in a maximum of −3 log10CFU decrease over 14 days. Drug B had similar activity compared to the standard regimen for drug‐susceptible TB (isoniazid, rifampicin, pyrazinamide, ethambutol, and HRZE), maximally resulting in a −2 log10CFU decrease over 14 days.^3^, ^19^ Drug C had a maximum activity of −1 log10CFU decrease over 14 days, similar to the overall activity of monotherapy of bedaquiline or delamanid.^20^, ^21^ Drug D had a similar maximum activity to drug B, but with a late onset of activity, after 7 days, reflecting a different mechanism of action, comparable to bedaquiline.

Two EC_50_s (AUC_0‐24_ leading to half of the *E*_max_) were investigated for each drug, reflecting the uncertainty in dose selection in relation to the potency of the drug in a phase IIa clinical trial. The low EC_50_ was set at 5.0 mg*h/L, below the average concentration reached by participants in the 1× q.d. dosing group, and the high EC_50_ was set at 25 mg*h/L, below the average concentrations reached by the 3× q.d. dosing group.

### Clinical trial simulations

In total, PK‐PD data for 1000 virtual patients, that is, 250 patients at each dosing level, were simulated for each hypothetical drug applying the *E*_max_ exposure‐response relationships. Duplicate measurements were simulated for each sampling timepoint. MCMP analyses were performed by analyzing CFU data, TTP data, or CFU and TTP data simultaneously to quantify the power to identify the exposure‐response relationship.^12^ Considering real‐world clinical trial feasibility, MCMP analysis was performed up to a maximum of 25 virtual patients per dose group. A full model with an *E*_max_ exposure‐response relationship (Slope_*E*max_) based on individual daily AUC_0‐24_ (Equation 2) was compared to a reduced model with a constant slope without an effect of drug exposure (Slope_reduced_; Equation 3). For all MCMP analyses, 10,000 replicates of each sample size were performed. Statistical significance was determined at *p* < 0.05 for 1 degree of freedom.

(3) $${\text{Slope}}_{\text{reduced},i}=\text{Typical slop}e_{i}*\left(1+\text{IIVslop}e_{i}\right)$$

where Slope_reduced_ represents model slopes in Equation 1 without an exposure‐response effect and *i* represents either the first or second slope.

### Misspecified exposure‐response relationship

We performed MCMP analyses on drug B with low EC_50_ to investigate the power to identify a linear exposure‐response relationship overlaying the data generated by the *E*_max_ model, for CFU, TTP, and the combined CFU + TTP data. A full model with a linear exposure‐response model (Slope_linear_) based on individual daily AUC_0‐24_ (Equation 4), was compared to a reduced model with a constant slope without an effect of drug exposure (Slope_reduced_) (Equation 3). MCMP power curves were generated up to 25 virtual participants per dose group.

(4) $${\text{Slope}}_{\text{linear},i}=\text{Typical slop}e_{i}*\left(1+\frac{\mathrm{AU}C_{0-24}}{\text{median}\left(\mathrm{AU}C_{0-24}\right)}\right)*E_{\mathrm{lin}}*\left(1+\text{IIVslop}e_{i}\right)$$

where Slope_Linear_ represents slopes following a linear exposure‐response relationship applied in Equation 1 and *i* represents either the first or second slope. *E*_lin_ represents the linear exposure‐response effect.

### Distribution of participants across dosing groups

To investigate the effect of uneven distribution of participants across dosing groups on the power obtained, 60 virtual participants were distributed across four dosing groups using five different strategies, detailed in Table S1. In short, a balanced scenario (15 participants per dosing group), a slightly unbalanced scenario (20 participants in the lowest and highest dosing groups), a highly unbalanced scenario (25 participants in the lowest and highest dosing group), a scenario with three dosing groups (20 participants per dosing group), and a negative control scenario (10 participants in the lowest and highest dosing groups) were postulated. MCMP analyses were performed using these scenarios for CFU, TTP, and the combined CFU + TTP model using drug B with low EC_50_.

### Sensitivity analysis

To investigate the robustness of our findings, we conducted a sensitivity analysis by varying the simulation parameters most likely influencing the power to identify an exposure‐response relationship. In total, 10 scenarios were investigated with drug B and low EC_50_ as reference (Table 1). The power of the model with 15 participants per dosing group was calculated for CFU, TTP, and CFU + TTP for each scenario.

**TABLE 1:** Sensitivity analysis scenarios analyzed.

Scenario	Parameter varied	Magnitude
1	IIV Slope 1 CFU IIV Slope 2 CFU	+50% +50%
2	IIV Slope 1 CFU IIV Slope 2 CFU	−50% −50%
3	IIV Slope 1 TTP IIV Slope 2 TTP	+50% +50%
4	IIV Slope 1 TTP IIV Slope 2 TTP	−50% −50%
5	Additive residual error CFU	+50%
6	Additive residual error CFU	−50%
7	Additive residual error TTP	+50%
8	Additive residual error TTP	−50%
9	Baseline negative culture chance CFU	+200%
10	Baseline negative culture chance TTP	+200%

Abbreviations: CFU, colony‐forming unit; IIV, interindividual variation; TTP, time‐to‐positivity.

### Software

Model development and MCMP analysis were performed using NONMEM 7.4 with PsN 5.0.0 (Perl‐speaks‐NONMEM), and Pirana 2.9.7 as the graphical interface.^22^, ^23^, ^24^ Data management and plot generation were performed in R 4.1.3 using RStudio 3.6.3 as an interface.^25^, ^26^

## RESULTS

### Pharmacodynamic model

In total, 99 participants were included in the model development, for which 1959 CFU and 1968 TTP measurements were available. One participant was excluded from the analysis due to only one positive culture sample being available. In total, 151 (7.7%) CFU samples and 22 (1.1%) TTP samples were culture‐negative. The baseline chance for CFU was determined at 6.4% and for TTP at 0.35%. An example code for the combined CFU + TTP model is provided in the Supplementary File S1.

**FIGURE 1:**
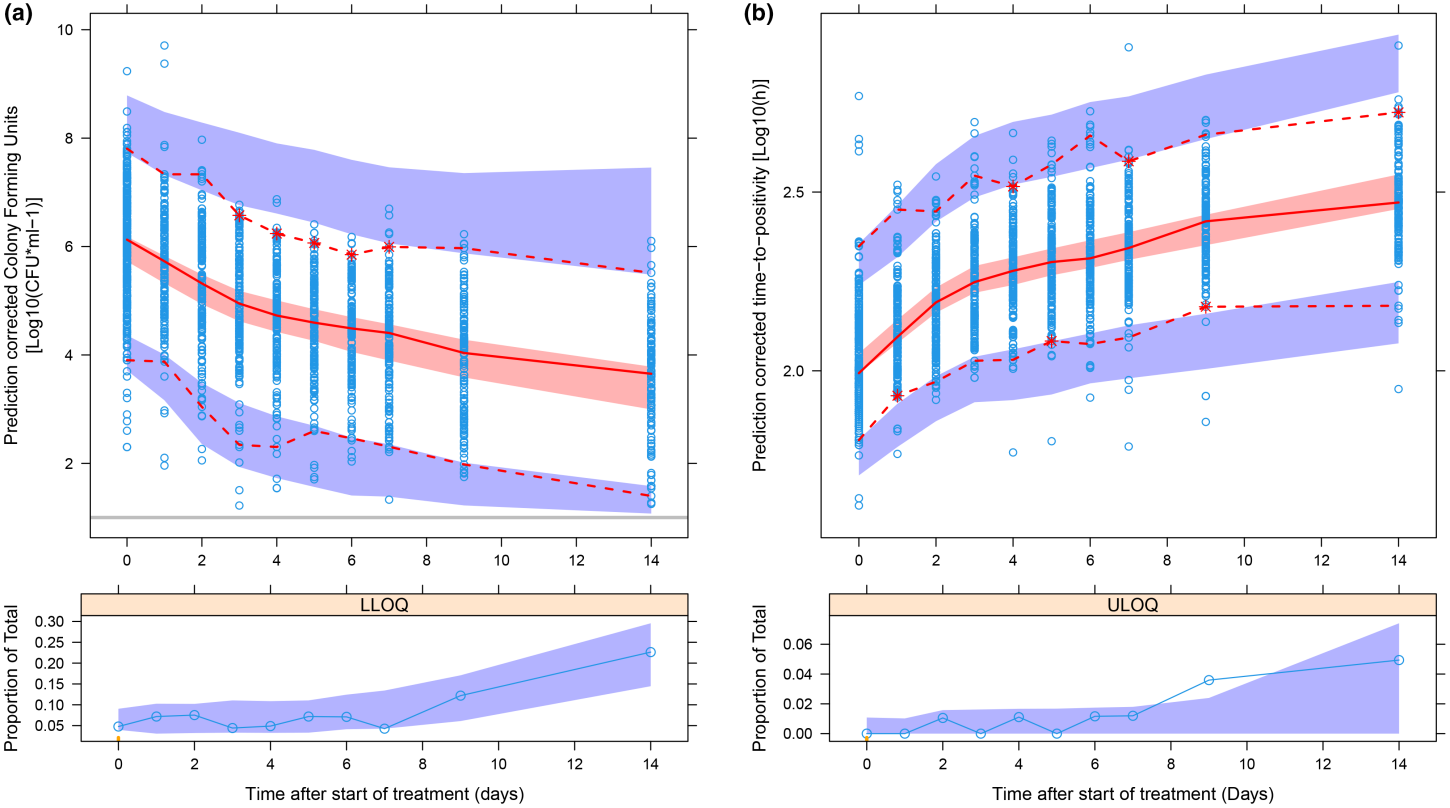
Prediction corrected visual predictive check showing the observed 2.5th, 50th, and 97.5th percentiles (lines) and 2.5th, 50th, and 97.5th prediction confidence intervals (shaded areas) from the combined CFU and TTP pharmacodynamic model. Left: Predicted CFU over time after the start of treatment, and percentage of predicted and observed lower limit of quantification observations in the lower panel. Right: Predicted TTP over time after the start of treatment, and percentage of predicted and observed upper‐limit of quantification observations in the lower panel. CFU, colony‐forming unit; TTP, time‐to‐positivity.

**FIGURE 2:**
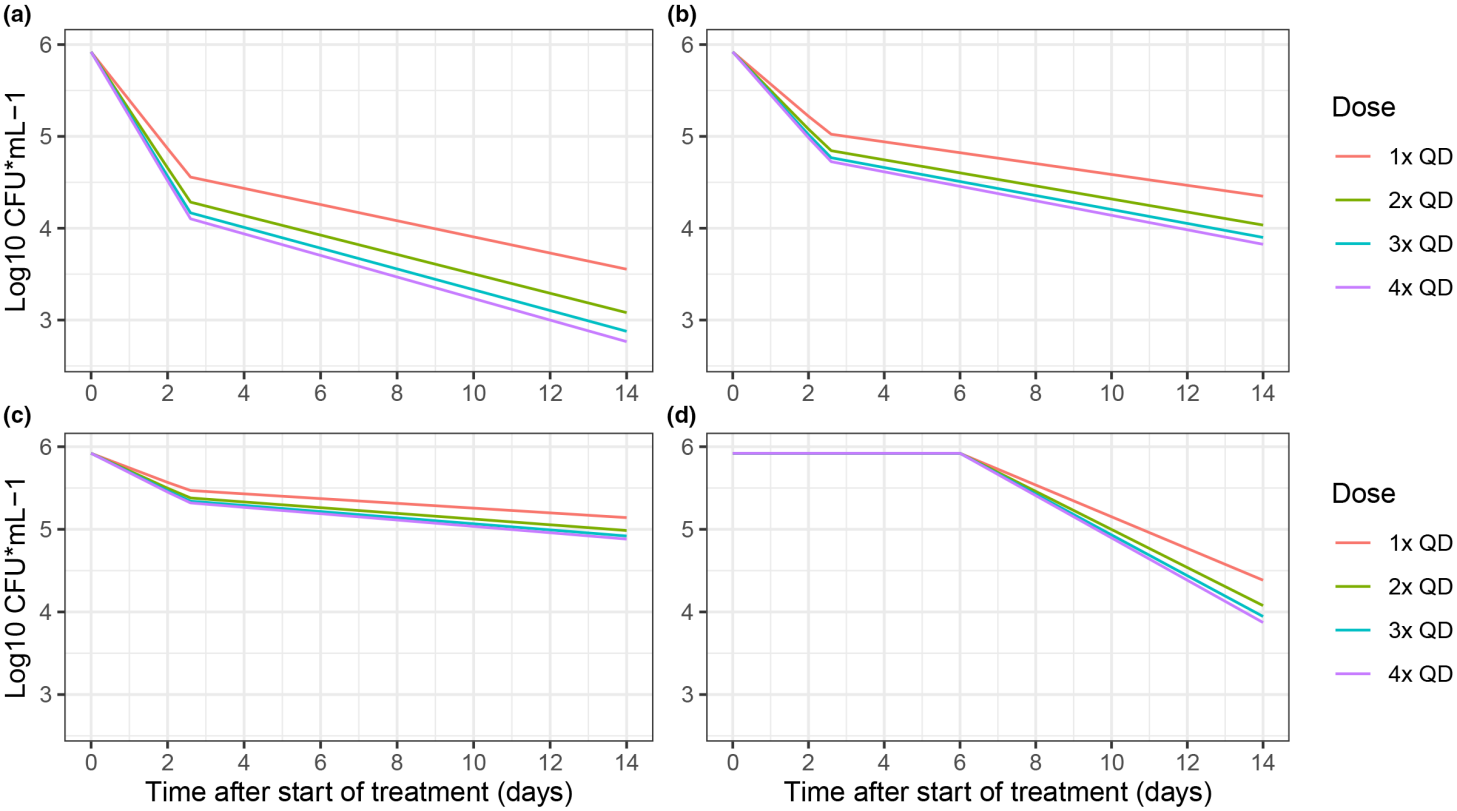
Typical simulated CFU measurements for 14 days after the start of treatment for the four hypothetical drugs (A–D) dosed daily in ratios of 1×, 2×, 3×, and 4×. CFU, colony‐forming unit; TTP, time‐to‐positivity.

The bilinear linear model with estimated node parameter on log10 transformed CFU and log10 transformed TTP data was selected as the best structural model. Final model parameters are reported in Table 2 and were used as input parameters for the clinical trial simulations. The sampling importance resampling (SIR) procedure was used to determine the parameter precision for the PD model.^27^ In total, six iterations were performed. SIR settings were 1000 samples and 500 resamples for the first five iterations, and 5000 samples and 500 resamples for the final iteration. Parameter precision was presented as 95% confidence intervals. Prediction‐corrected visual predictive checks for the final combined CFU + TTP PD model are provided in Figure 1. Typical simulated CFU profiles for each hypothetical drug at low EC_50_ are presented in Figure 2. Simulated typical CFU and TTP profiles for each hypothetical drug are available in Figure S1.

**TABLE 2:** Estimated model parameters and parameters used in the simulation model.

	Estimate (SIR 95% CI)	Simulation value
Fixed effects		
Baseline CFU (log10 CFU*mL^−1^)	5.92 (5.75–6.08)	5.92
Baseline TTP (log10h)	2.02 (1.99–2.04)	2.02
Slope 1 CFU (log10 CFU*mL^−1^*h^−1^)	0.0141 (0.0121–0.0162)	Max *E*_max_ slope: A: 0.0329 B: 0.0219 C: 0.0110 D: 0
Slope 1 TTP (−log10h*h^−1^)	0.00353 (0.00320–0.00391)	Max *E*_max_ slope: A: 0.0056 B: 0.0037 C: 0.0019 D: 0
Slope 2 CFU (log10 CFU*mL^−1^*h^−1^)	0.00662 (0.00552–0.00743)	Max *E*_max_ slope: A: 0.00550 B: 0.00370 C: 0.00180 D: 0.0120
Slope 2 TTP (−log10h*h^−1^)	0.000910 (0.000810–0.00100)	Max *E*_max_ slope: A: 0.00093 B: 0.00063 C: 0.0003 D: 0.00203
Node (h)	62.1 (56.3–67.7)	A, B, C: 62.1 D: 144.0
Baseline chance negative culture CFU (%)	6.40	6.40
Baseline chance negative culture TTP (%)	0.35	0.35
Clearance (L/h)	Not applicable	10.0
Volume of distribution (L)	Not applicable	40.0
EC_50_ (mg*h/L)	Not applicable	Low:5.0 High:25.0
Random effects		
IIV baseline CFU (CV%)	15.4 (13.4–18.3)	15.4
IIV baseline TTP (CV%)	5.5 (4.9–6.1)	5.5
Correlation baseline CFU‐baseline TTP (%)	−71.5 (−100.0 to −38.8)	−71.5
IIV slope 1 CFU (CV%)	58.3 (48.5–70.6)	58.3
IIV slope 1 TTP (CV%)	30.9 (24.3–36.1)	30.9
Correlation slope 1 CFU‐slope 1 TTP (%)	94.1 (92.3–100.0)	94.1
IIV slope 2 CFU (CV%)	74.1 (62.7–88.8)	74.1
IIV slope 2 TTP (CV%)	47.2 (38.3–57.6)	47.2
Correlation slope 2 CFU‐slope 2 TTP (%)	63.4 (54.2–69.3)	63.4
IIV clearance (CV%)	Not applicable	30.0
Additive error CFU (log10 CFU*mL^−1^)	0.334 (0.291–0.360)	0.334
Additive error TTP (log10h)	0.00667 (0.00606–0.00730)	0.00667
Correlation additive error CFU‐additive error TTP (%)	−47.4 (−63.7 to −37.9)	−47.4
Correlation CFU replicate error (%)	90.3 (89.7–90.5)	90.3
Correlation TTP replicate error (%)	82.7 (81.4–83.2)	82.7

*Note*: CV% was calculated as sqrt(exp(OMEGA^2^) − 1).

Abbreviations: CFU, colony‐forming unit; CI, confidence interval; EC_50_, half‐maximal effective concentration; *E*_max_, maximum effect; IIV, interindividual variation; SIR, sampling importance resampling; TTP, time‐to‐positivity.

### Clinical trial simulations

The overall power to identify the exposure‐response relationship was low for drugs with moderate activity (C) or late onset of activity (D), irrespective of the data analyzed. The power to identify an exposure‐response relationship for drug C was below 60% for each data type analyzed. An exposure‐response relationship of drug D was identifiable with more than 80% power for CFU, TTP, and combined data, but only for a hypothetical drug with a high EC_50_. Overall power to identify the exposure‐response of the hypothetical drugs with high activity (A and B) was greater than 80% for all types of data investigated except for CFU in combination with a low EC_50_.

**FIGURE 3:**
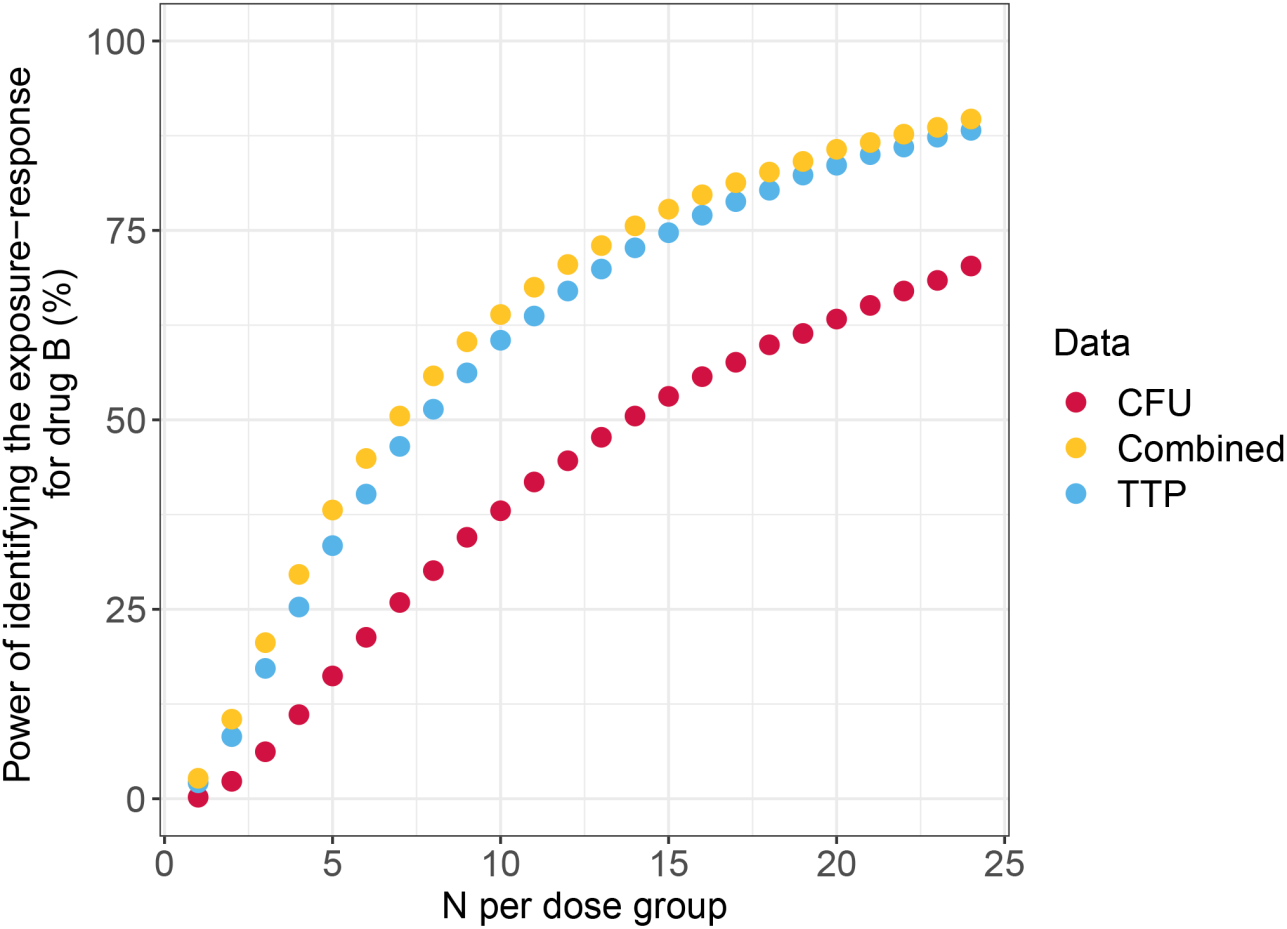
Power of detecting an exposure‐response relationship using the CFU, TTP, or combined CFU and TTP models at different dosing group sizes for drug B with a low EC_50_. CFU, colony‐forming unit; EC_50_, half‐maximal effective concentration; IIV, interindividual variation; TTP, time‐to‐positivity.

CFU data provided the model with low power to identify an exposure‐response model for most drugs. At 60 total participants, CFU data analysis was not able to identify an exposure‐response relationship with over 80% power for all drugs with a low EC_50_. Analysis of TTP data improved power compared to CFU for all investigated drugs. On average (range), the power analyzing TTP data was 10.9% (1.9%–29.4%) higher compared to analyzing the CFU data. Combined analysis of CFU and TTP data further improved the power over the TTP data analysis. The effect was modest; on average (range), the power increased by 4.2% (0%–12.8%). Drug C (with the lowest overall effect) showed the largest increase in power using a combined analysis of CFU + TTP compared to the analysis of TTP with an increase in power of 12.8% and 7.4% for low and high EC_50_, respectively. For drug B with low EC_50_, the total number of participants needed to reach an 80% power was 136 for CFU, 72 for TTP, and 68 for combined CFU + TTP analysis. MCMP results for all hypothetical drugs are presented in Table 3. The CFU, TTP, and combined CFU + TTP power curve for drug B with low EC_50_ is presented in Figure 3. MCMP power curves for all drugs are presented in Figure S2.

**TABLE 3:** Power to identify an exposure‐response relationship using CFU, TTP, or combined CFU + TTP data at *n* = 15 per dosing group.

Drug	Power using CFU (%)	Power using TTP (%)	Power using combined CFU + TTP (%)
A (low EC_50_)	55.8	85.2	87.9
B (low EC_50_)	53.1	74.7	77.8
C (low EC_50_)	24.5	31.8	44.6
D (low EC_50_)	48.1	51.2	55.9
A (high EC_50_)	98.1	100	100
B (high EC_50_)	93.1	97.3	98.3
C (high EC_50_)	38.1	51.2	58.6
D (high EC_50_)	87.2	93.9	95.6

Abbreviations: CFU, colony‐forming unit; EC_50_, half‐maximal effective concentration; TTP, time‐to‐positivity.

The choice of doses selected in relation to the EC_50_ was shown to have a large impact on the power to identify an exposure‐response relationship. The difference in power between all investigated drugs with a low EC_50_, and all investigated drugs with a high EC_50_ was, on average (range), 26.7% (12.6%–42.7%).

### Misspecified exposure‐response relationship

The linear model had a slightly lower power to identify an exposure‐response relationship compared to using the *E*_max_ model when the *E*_max_ model was used to simulate the analyzed data. The difference in power was more pronounced when analyzing TTP data or combined CFU and TTP data, compared with analyzing CFU data alone. At 15 participants per dosing group, the linear model showed 1.4% lower power than the *E*_max_ model for CFU, 10.2% lower power for TTP, and 10.0% lower power for CFU + TTP combined analysis. The full MCMP power curves for detecting a linear exposure‐response relationship for drug B with low EC_50_ when using CFU, TTP, or combined CFU + TTP, compared to detecting an *E*_max_ exposure‐response relationship are presented in Figure S3.

### Distribution of participants across dosing groups

Improved power was observed for scenarios with more participants distributed to the extreme dose groups. The highly unbalanced design (25 participants in the lowest and highest dosing groups) improved the power over the balanced scenario by 16% at 60 total participants. The slightly unbalanced scenario (20 participants in the lowest and highest dosing groups) and using three instead of four arms performed comparably, with an increase in power over the balanced design of 7.3% and 6.4%, respectively, at 60 total participants. MCMP power curves for scenarios 1–5 are presented in Figure S4.

### Sensitivity analysis

**FIGURE 4:**
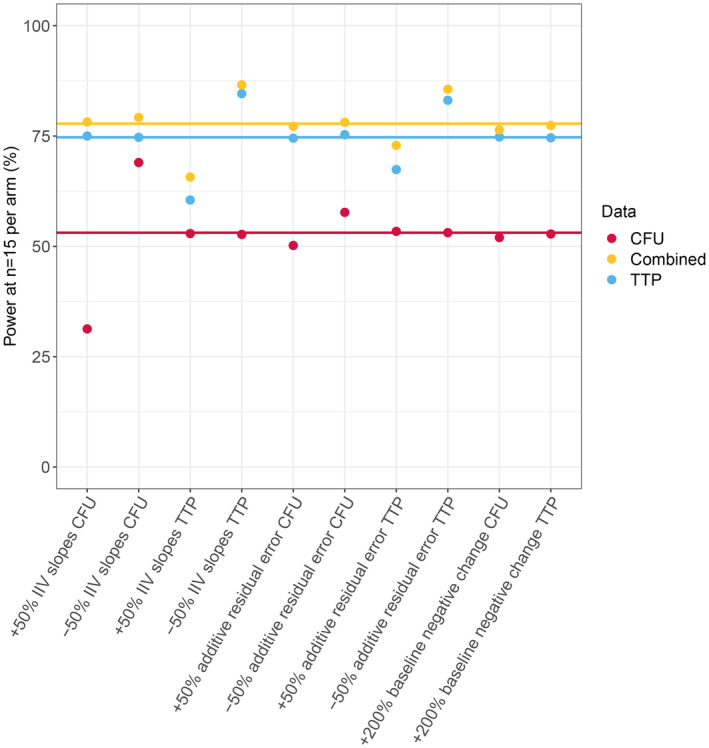
Power to identify an *E*_max_ exposure‐response relationship for CFU (red), TTP (blue), and combined CFU and TTP (yellow) for sensitivity analysis scenarios 1 through 10 of drug B with low EC_50_. Horizontal lines represent the power of the reference model (drug B with low EC_50_ without any model parameters varied from the simulation model). CFU, colony‐forming unit; EC_50_, half‐maximal effective concentration; LLOQ, lower limit of quantification; TTP, time‐to‐positivity; ULOQ, upper limit of quantification.

In all sensitivity analyses performed, combined CFU + TTP data analysis performed best, as the power to identify an exposure‐response relationship at 15 participants per group was higher compared to TTP or CFU data analysis alone. Furthermore, TTP data analysis provided the model more power to identify an exposure‐response relationship compared to CFU in all investigated sensitivity scenarios. The difference in power with the reference was most affected by the IIV of the slopes. The impact of additive residual error power was less pronounced. Baseline negative culture chance did not have a large impact on the power obtained for all data types. The estimated power to identify an exposure‐response relationship for the sensitivity analysis scenarios is presented in Figure 4 for CFU, TTP, and combined CFU + TTP data analysis.

## DISCUSSION

Our study demonstrates the low overall power to identify an exposure‐response relationship for drugs with low overall activity or slow onset of activity in a phase IIa clinical trial for TB. The hypothetical drug that was most similar to monotherapy of delamanid or bedaquiline, showed low power (<59%), irrespective of the analysis of CFU, TTP, or combined CFU + TTP data. Current EBA trials, including 60 participants divided evenly across four dosing groups, might therefore be underpowered to detect an exposure‐response relationship.

Analysis of CFU data provided the model with relatively low power to identify the exposure‐response relationship. The power to identify an exposure‐response relationship was improved when analyzing TTP data compared to CFU data. Analyzing both CFU + TTP in a combined approach further provided the model with a modest improvement in power compared to only analyzing TTP data. Whereas we had anticipated a larger effect, the added value of using a combined CFU and TTP data analysis approach over only analyzing TTP data was modest for most investigated drugs. The highest gain in overall power was observed for drugs with a low bactericidal effect, although drugs with a higher activity gained less power from the combined data analysis. Although not the primary objective of an EBA, additional early knowledge can be gained by powering EBA studies to evaluate PKs‐PDs, which may better inform later phase trials focused on dose‐finding, and accelerate the drug development timeline.

The distribution of investigated doses in relation to the EC_50_ of a drug is expected to impact the power to identify an exposure‐response model strongly. We have shown that drugs with an EC_50_ corresponding to the median exposure in the 3× dosing group had a considerably higher power compared to drugs with an EC_50_ corresponding to the median exposure in the 1× dosing group. When the EC_50_ is low in relation to the exposures generated by the investigated doses, a large part of the data is collected in the range corresponding to maximal effect which decreases the ability to describe the relationship. Translational predictions based on preclinical data, together with population PK and safety data from phase I clinical trials, should be used to select relevant doses for EBA studies to maximize statistical power under the given sample size. Furthermore, analysis choices such as the type and parameterization of the exposure‐response model may have an impact on the power obtained. Our results show that the difference in power when trying to identify a linear versus an *E*_max_ exposure‐response model is limited. In practice, both a linear and *E*_max_ model might be explored, based on the available data. The *E*_max_ model in our study was parameterized to have one parameter more compared to the model without a drug effect. This model assumes no decrease in bacterial load in the absence of exposure, and might therefore only be applicable in trials where monotherapy of the investigated drug is the only driver of the antimicrobial effect. For studies investigating a combination drug regimen, one would need to expand the *E*_max_ model with at least one more parameter, representing the bactericidal effect of the background drugs, which would likely decrease the power further.

Our study highlights the importance of clinical trial design for secondary end points in EBA studies. Distributing more participants to the highest and lowest dosing groups substantially improved the power to identify an exposure‐response relationship. Such unbalanced designs might be applicable to some phase IIa studies, whereas in dose‐finding studies it might be more difficult to implement. Another objective of phase II clinical trials is the early assessment of drug safety or tolerability, distributing fewer participants to certain dosing groups might impact the safety assessment. Other design choices such as the timing of the sputum samples, amount of sputum samples, and trial duration might also have an impact on the power to identify an exposure‐response relationship.

The PD model used for simulations in our study was developed on data from one clinical trial. Even though phase IIa studies are very standardized, data from these studies are highly variable, primarily due to the substantial inter‐ and intra‐patient variability.^9^ Therefore, other studies might find differences in parameter estimates and, by extent, different power to identify an exposure‐response relationship. Our sensitivity analysis shows that our findings are robust, as the combined data analysis performed best in all sensitivity analysis scenarios investigated, and the TTP‐only data analysis had a higher power to identify exposure‐response relationships compared to the CFU‐only data analysis. The IIV in the slope was identified as the main model parameter influencing the power, stressing the importance of variability reduction measures in these trials. This result is consistent with a previous study that found that the sample size needed to achieve 80% power to detect a difference in EBA between two groups increased with increasing IIV on the TTP slope.^28^

We assumed the exposure‐response relationship was of a similar magnitude for CFU and TTP during the entire 14‐day clinical trial period. In practice, different drugs might have a different impact on CFU or TTP, and their impact might also change over time. This may reflect the different activity of the drugs or sensitivities of the culture method for TB subpopulations, or changes in metabolic rates of the TB mycobacteria due to treatment.^29^ However, during model development, no significant difference was found when estimating separate exposure‐response relationships for CFU and TTP on HIGHRIF1 data. Compounds with a different mechanism of action than rifampicin might influence the exposure‐response relationship differently for CFU and TTP, and might therefore influence the power obtained.

The PD model was developed on the assumption that the time after treatment initiation at which the slope parameter changes were similar for CFU and TTP. This was consistent with our findings in the separate models where the node of rate switch for the CFU models was estimated to be at 59 and 62 h for the TTP model. This finding might not be applicable to all drugs, complicating the development of a combined CFU and TTP model.

Mockeliunas et al.^28^ performed an in silico study using a linear model to determine the power to detect EBA, and the power to identify a difference in EBA between two treatment groups in phase IIa trials using TTP‐based analysis. The authors reported that a sample size of 15 participants was deemed sufficient for detecting EBA. However, their findings further indicated that when comparing a drug with similar activity to drug C in this study (1 logCFU decline over 14 days) to a drug with 50% increased activity relative to drug C, TTP‐based analysis may lack statistical power to discern the difference at 15 participants per group. Conversely, when comparing a drug with an activity profile akin to drug B in this study (2 log CFU decline over 14 days) to a drug with 50% increased activity relative to drug B, a sample size of 15 participants was generally sufficient. Our results complement these findings by also demonstrating that statistical power is also be lacking to identify an exposure‐response for drug C while generally being adequate for drug B.

In conclusion, our study shows that a TTP‐based model has higher power to identify exposure‐response relationships compared to CFU, and combined CFU + TTP model analysis slightly further improves the power. The power to identify an exposure‐response relationship is low for TB drugs with moderate bactericidal activity or slow onset. Utilizing unbalanced sample size designs in clinical trials may improve the power further, increasing certainty in dose selection for further clinical trials.

## AUTHOR CONTRIBUTIONS

S.E.K., T.P.C.D., C.M.U., R.E.A., and E.M.S. wrote the manuscript. S.E.K., and E.M.S. designed the research. S.E.K. performed the research. S.E.K., T.P.C.D., C.M.U., R.E.A., and E.M.S. analyzed the data.

## FUNDING INFORMATION

The work for this publication was partly funded by a Veni project (EM Svensson, project number 09150161910052) financed by the Dutch Research Council (NWO).

## CONFLICT OF INTEREST STATEMENT

The authors declare no competing interests in this study.

## Supporting information


Data S1.
