# Supplementary material for: Power to identify exposure‐response relationships in phase IIa pulmonary tuberculosis trials with multi‐dimensional bacterial load modeling
Source: CPT Pharmacometrics Syst Pharmacol. 2023 Dec 15;13(3):374–85. doi: 10.1002/psp4.13089 (PMC10941589; doi:10.1002/psp4.13089)
Supplement: Supplementary file 1 — Data S1. [file PSP4-13-374-s001.docx]

**Supplementary files:** **Power to identify exposure-response relationships in phase IIa pulmonary tuberculosis trials with multi-dimensional bacterial load modeling.**

Simon E. Koele^1^, Thomas P.C. Dorlo^2^, Caryn M. Upton^3^, Rob Aarnoutse^1^, Elin M. Svensson^1,2^

^1^ Department of Pharmacy, Radboudumc Research Institute for Medical Innovation (RIMI), Radboud university medical center, Nijmegen, the Netherlands.

^2^ Department of Pharmacy, Uppsala University, Uppsala, Sweden.

^3^ TASK Applied Science, Cape Town, South Africa.

***Table S1:*** *Investigated dosing group sizes for the five investigated clinical trial design strategies. N per group represents the number of participants receiving the corresponding dose of that group.*

| **Scenario/Dose arm** | **Dose 1x (n)** | **Dose 2x (n)** | **Dose 3x (n)** | **Dose 4x (n)** | **Total (n)** |
| --- | --- | --- | --- | --- | --- |
| Balanced | 15 | 15 | 15 | 15 | 60 |
| Slightly unbalanced | 20 | 10 | 10 | 20 | 60 |
| Highly unbalanced | 25 | 5 | 5 | 25 | 60 |
| Three dosing groups | 20 | 20 | 0 | 20 | 60 |
| Negative control | 10 | 20 | 20 | 10 | 60 |

*
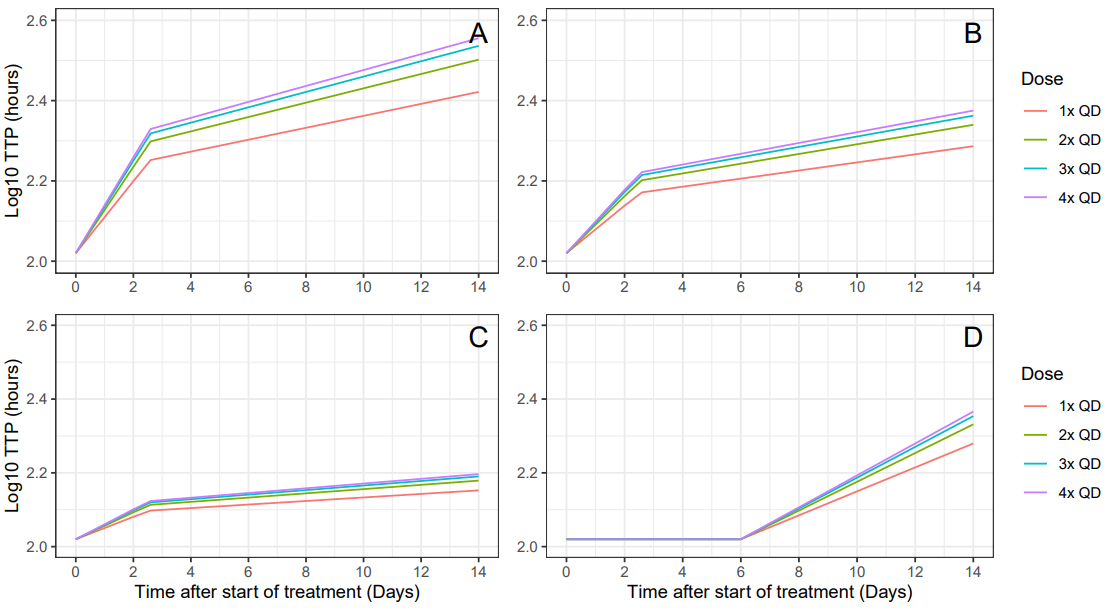
*

***Figure S1a:*** *Typical simulated TTP measurements for 14 days after the start of treatment for the four hypothetical drugs (A-D) with low EC_50_ dosed daily in ratios of 1x, 2x, 3x, and 4x.*

*
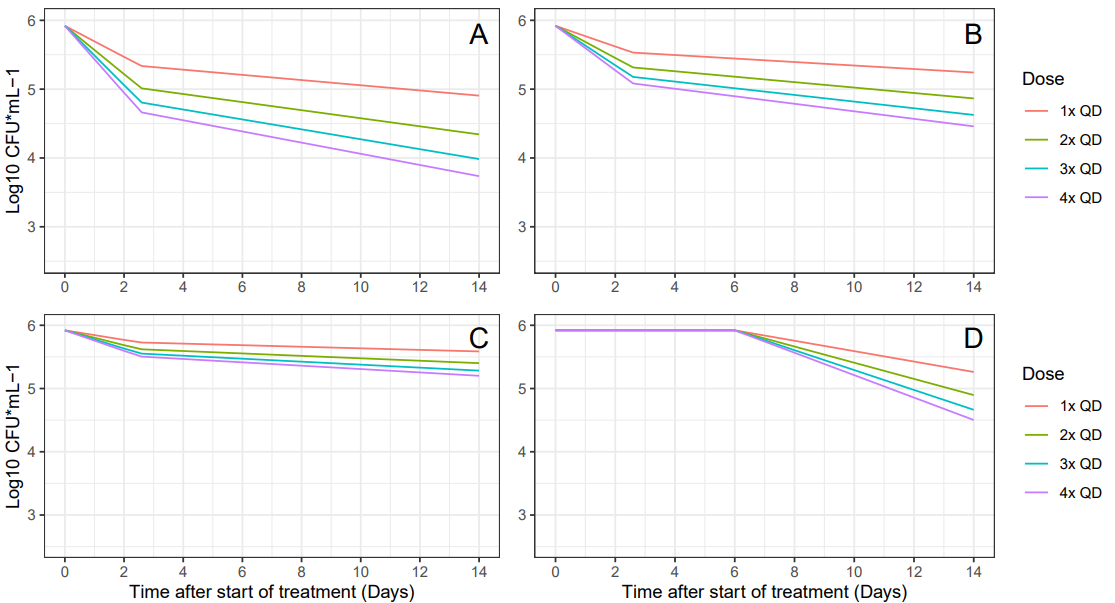
*

***Figure S1b:*** *Typical simulated CFU measurements for 14 days after the start of treatment for the four hypothetical drugs (A-D) with high EC_50_ dosed daily in ratios of 1x, 2x, 3x, and 4x.*

*
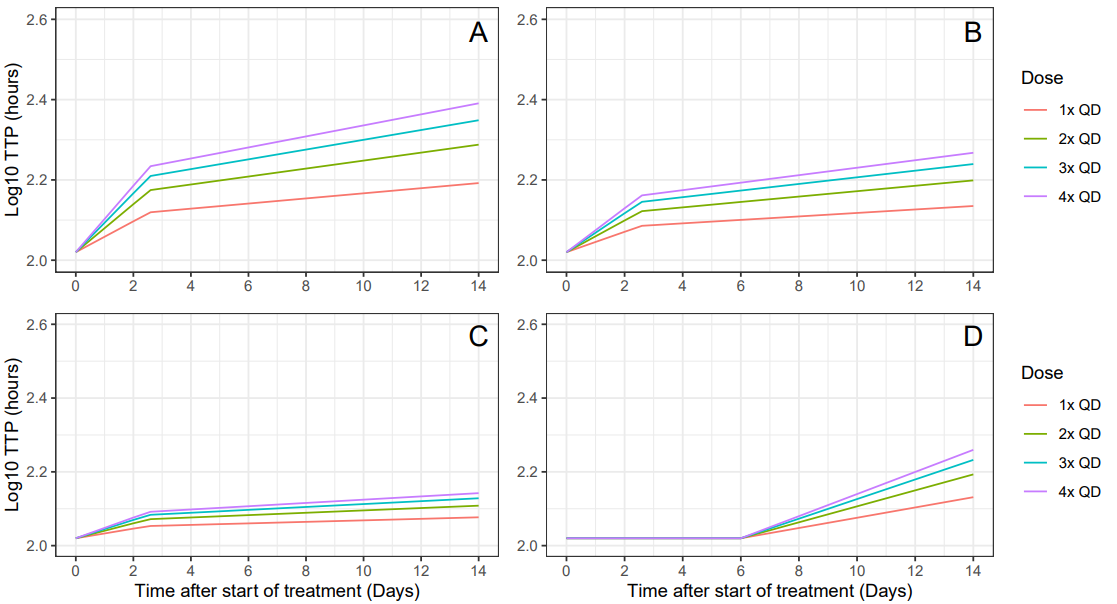
*

***Figure S1c:*** *Typical simulated TTP measurements for 14 days after the start of treatment for the four hypothetical drugs (A-D) with high EC_50_ dosed daily in ratios of 1x, 2x, 3x, and 4x.*


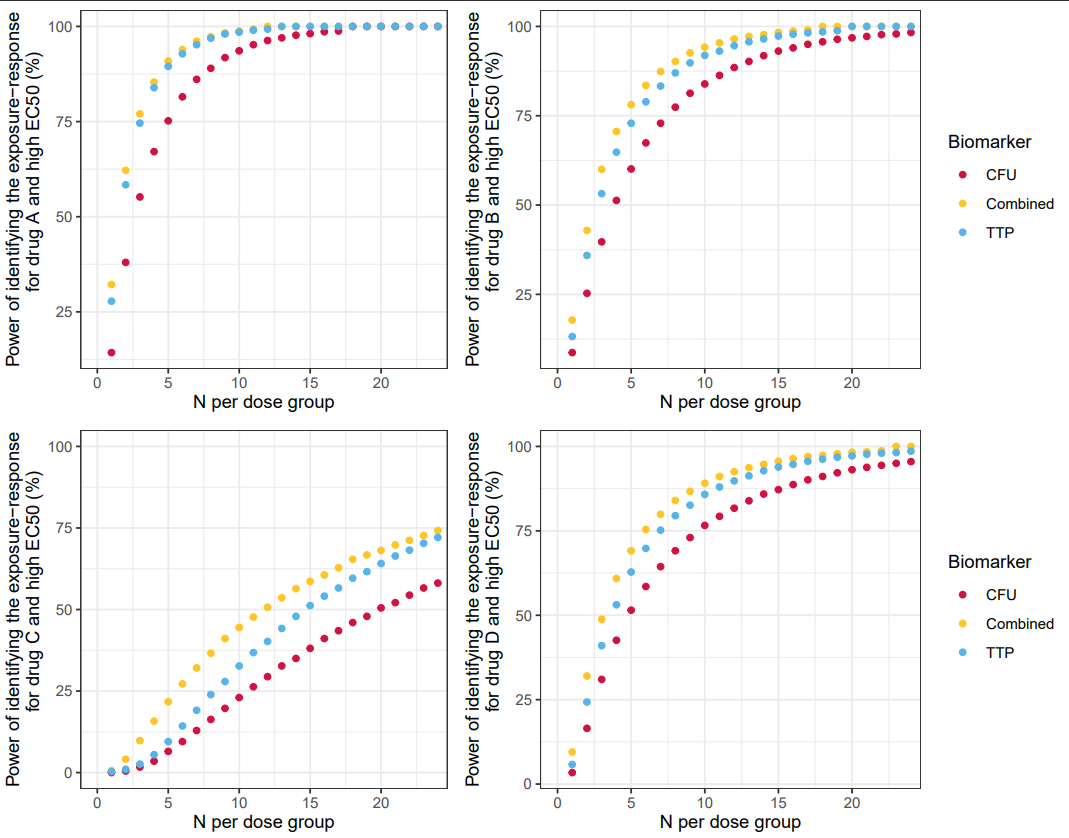


***Figure S2a:*** *Power of detecting an exposure-response relationship using the CFU, TTP, or combined CFU and TTP models at different dosing group sizes for drugs with a high EC_50_.*

*
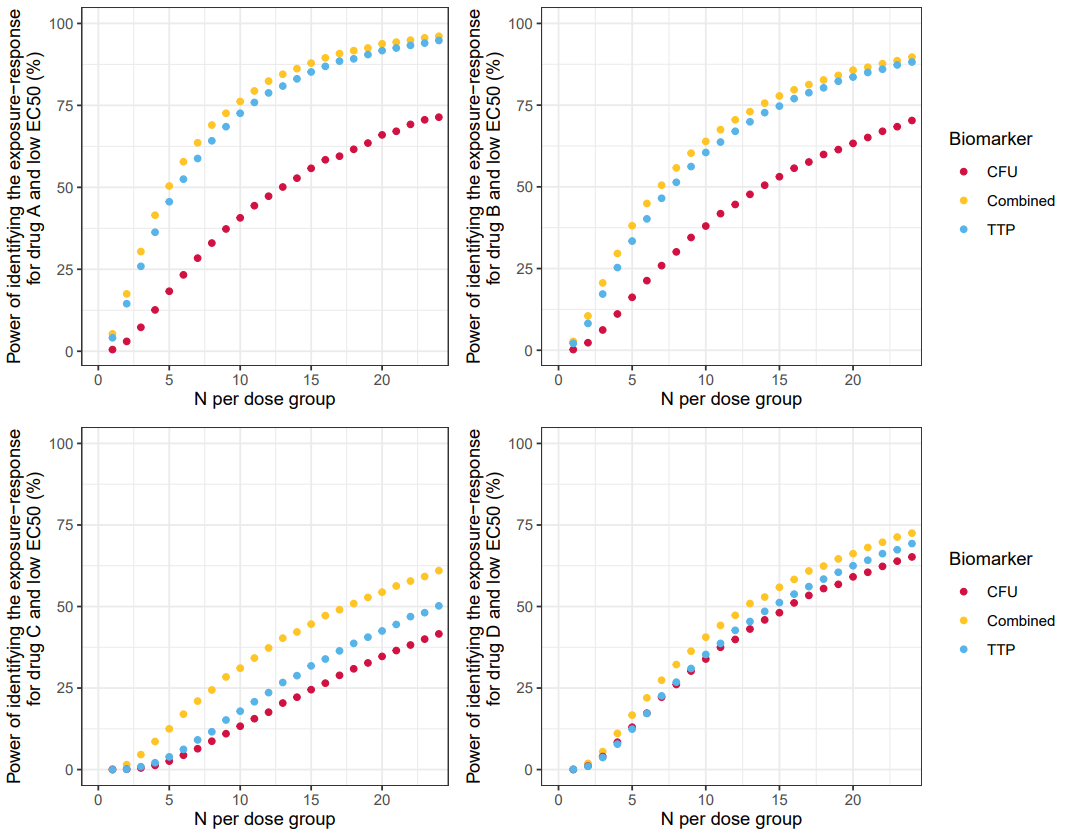
*

***Figure S2b:*** *Power of detecting an exposure-response relationship using the CFU, TTP, or combined CFU and TTP models at different dosing group sizes for drugs with a high EC_50_.*


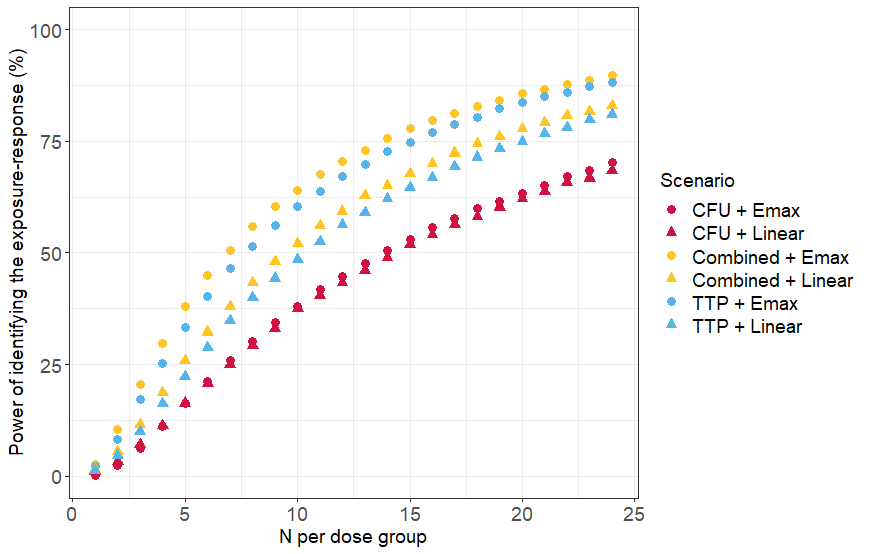


***Figure S3:*** *MCMP power curves for drug B using a linear model for exposure-response (triangles) or an Emax model (circles). Yellow represents the combined models, red the CFU models, and blue the TTP models.*


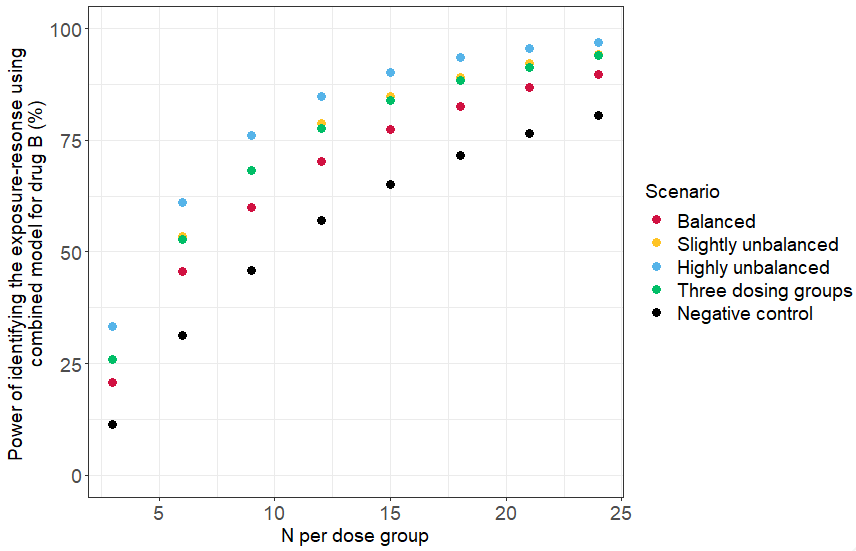


***Figure S4:*** *Combined analysis of CFU+TTP MCMP power curve for balanced (red), slightly unbalanced (yellow), highly unbalanced (blue), using three dosing groups (green), and negative control (black) study designs for drug B with low EC50.*

**Example code for NONMEM 7.4 combined CFU+TTP model**

$PROB CFU+TTP exposure response

$INPUT ID TIME DV FLAG EVID MDV TYPE REP EXMET L2

;ID = Subject ID

;TIME = Time after start treatment (h)

;DV = Bacterial load measurements

;FLAG = 1/2 for CFU or TTP respectively

;EVID = Event ID

;MDV = Missing dependent variable

;TYPE = 1/2 for continuous observations or negative culture result respectively

;REP = 1/2 for first and second measurement at replicate timepoint respectively

;EXMET = Exposure metric to test for exposure-response identification

;L2 = L2 data item

$DATA … IGNORE =@

$PRED

;---------------------------- Parameters for bacterial load model ---------------------------------

INTERCEPTCFU = THETA(1) *EXP(ETA(1))

INTERCEPTTTP = THETA(2) *EXP(ETA(2))

EMAXCFU1= THETA(3)

EMAXCFU2= THETA(4)

EC50CFU= THETA(5)

EFFBETA1CFU= (EMAXCFU1*EXMET)/(EC50CFU+ EXMET)

EFFBETA2CFU= (EMAXCFU2* EXMET)/(EC50CFU+ EXMET)

EMAXTTP1= THETA(6)

EMAXTTP2= THETA(7)

EC50TTP= THETA(5)

EFFBETA1TTP= (EMAXTTP1* EXMET)/(EC50TTP+ EXMET)

EFFBETA2TTP= (EMAXTTP2* EXMET)/(EC50TTP+ EXMET)

BETA1CFU = EFFBETA1CFU *EXP(ETA(3))

BETA2CFU = EFFBETA2CFU *EXP(ETA(5))

BETA1TTP = EFFBETA1TTP *EXP(ETA(4))

BETA2TTP = EFFBETA2TTP *EXP(ETA(6))

NODE = THETA(8)

;---------------------------- Bacterial load calculation -----------------------------------------

CFU1= INTERCEPTCFU - BETA1CFU*TIME

CFUatNODE= INTERCEPTCFU - BETA1CFU*NODE

CFU2= CFUatNODE - BETA2CFU*(TIME-NODE)

TTP1= INTERCEPTTTP + BETA1TTP*TIME

TTPatNODE= INTERCEPTTTP + BETA1TTP*NODE

TTP2= TTPatNODE + BETA2TTP*(TIME-NODE)

CFU = CFU1

IF (TIME.GT.NODE) CFU= CFU2

TTP = TTP1

IF (TIME.GT.NODE) TTP= TTP2

IF(FLAG.EQ.1) IPRED = CFU

IF(FLAG.EQ.2) IPRED = TTP

;------------------------------ Probability of bacterial presence -------------------

IF (FLAG.EQ.1) PBAC = 0.064 ; Impute chance of obtaining negative culture result CFU during first 3 days of treatment

IF (FLAG.EQ.2) PBAC = 0.0035 ; Impute chance of obtaining negative culture result TTP during first 3 days of treatment

;Additive error for each replicate of the same sputum sample

IF (FLAG.EQ.1.AND.REP.EQ.1) ADDERR = EPS(1)

IF (FLAG.EQ.2.AND.REP.EQ.1) ADDERR = EPS(2)

IF (FLAG.EQ.1.AND.REP.EQ.2) ADDERR = EPS(3)

IF (FLAG.EQ.2.AND.REP.EQ.2) ADDERR = EPS(4)

;----------------------- M3 method for handling negative samples ------------------------

IF (FLAG.EQ.1) SD = SQRT(SIGMA(1,1))

IF (FLAG.EQ.2) SD = SQRT(SIGMA(2,2))

LLOQ=1 ;LLOQ for CFU

ULOQ=3.003641 ;ULOQ for TTP

DUMLLOQ=(LLOQ-IPRED)/SD

DUMULOQ=(IPRED-ULOQ)/SD

IF (FLAG.EQ.1) CUMD=PHI(DUMLLOQ)

IF (FLAG.EQ.2) CUMD=PHI(DUMULOQ)

IF(ICALL.EQ.4) TYPE=2

IF (TYPE.EQ.2) THEN

F_FLAG=0

Y=IPRED + ADDERR

IRES = DV - IPRED

ENDIF

IF (TYPE.EQ.1) THEN

F_FLAG=1

Y=CUMD+PBAC-(CUMD*PBAC)

MDVRES = 1

ENDIF

$THETA

(0,5) ; 1 Intercept CFU

(0,2) ; 2 Intercept TTP

(0,0.01,1) ; 3 EMAXCFU1

(0,0.004,1) ; 4 EMAXCFU2

(0,0.001,1) ; 5 EMAXCTTP1

(0,0.0003,1) ; 6 EMAXTTP2

(0,25) ; 7 EC50

(0,50,150) ; 8 NODE

$OMEGA BLOCK(2)

0.1; 1 intercept CFU

0.01 0.1; 2 intercept TTP

$OMEGA BLOCK(2)

0.1; 3 slope1 CFU

0.01 0.1; 4 slope1 TTP

$OMEGA BLOCK(2)

0.1; 5 slope2 CFU

0.01 0.1; 6 slope2 TTP

$SIGMA BLOCK(4)

0.1 ; 1 additive error CFU replicate1

0.01 0.1 ; 2 additive error TTP replicate 1

0.01 0.01 0.1; 3 additive error CFU replicate 2

0.01 0.01 0.01 0.1; 4 additive error TTP replicate 2

$ESTIM METHOD=1 INTERACTION LAPLACIAN NUMERICAL SLOW MAXEVAL=9999 PRINT=1

$COV UNCONDITIONAL

$TABLE ….
